# Factors associated with regular counselling attendance of HIV outpatients of a national referral hospital in Jakarta, Indonesia: a cross sectional study

**DOI:** 10.1186/s12889-018-5924-5

**Published:** 2018-08-20

**Authors:** M. Pane, E. I. Sianturi, Yin Mei Fiona Kong, Philip Bautista, Herlina, K. Taxis

**Affiliations:** 1 Sulianti Saroso Infectious Diseases Hospital, Ministry of Health, Jakarta, Republic of Indonesia; 2 PharmacoTherapy, -Epidemiology & -Economics (PTEE), Groningen Research Institute of Pharmacy, University of Groningen, Antonius Deusinglaan 1, 9713 AV Groningen, The Netherlands; 3 Faculty of Mathematics and Natural Sciences, University of Cenderawasih, Jayapura, Papua Indonesia; 4 The University of Hong Kong, Hongkong, China; 5 Independent researcher, Washington, DC, USA

**Keywords:** Counselling, Adherence, PLHIV, Indonesia

## Abstract

**Background:**

Counselling has been shown to improve adherence to medication in people living with HIV (PLHIV). The aim of this study was to investigate factors associated with regular counselling attendance of patients taking antiretroviral therapy (ART).

**Methods:**

We conducted a cross-sectional, paper-based survey among 880 PLHIV patients on ART attending outpatient clinics of a referral hospital in Jakarta. Patients on ART, above 18 years old, providing written consent were included. The primary outcome was regular counselling attendance (i.e., having attended at least 3 sessions in the previous 3 months) using records from counsellors. Factors associated with regular counselling attendance were assessed using logistic regression analysis.

**Results:**

The majority of patients were male (71.1%) and had regular counselling (78.4%). Being 31 to 40 years old (odds ratio (OR) = 0.55, 95% confidence interval (CI) = 0.32–0.93, > 40 years (OR = 0.30, 95% CI = 0.16–0.55) vs < 30 years, hepatitis B/C co-infection (OR = 0.42, 95% CI = 0.24–0.75), living > 20 km from the hospital (OR = 0.55, 95% CI = 0.33–0.93), transmission male-to-male (OR = 0.13, 95% CI = 0.04–0.44), unemployment (OR = 1.88, 95% CI = 1.02–3.44), part-time employment (OR = 10.71, 95% CI = 4.09–28.02), household member with HIV (OR = 3.31, 95% CI = 1.70–6.44), and Christianity (OR = 1.82, 95% CI = 1.12–2.94) were associated with regular counselling attendance.

**Conclusion:**

This study suggests that counselling services should be reviewed to ensure that they are near home and fit the needs of older patients or patients with co-morbidities and minorities. Tailoring counselling may improve attendance.

**Electronic supplementary material:**

The online version of this article (10.1186/s12889-018-5924-5) contains supplementary material, which is available to authorized users.

## Background

Indonesia, the fourth most populated country in the world, reported a five-fold increase in human immunodeficiency virus (HIV)-infected individuals from 2001 to 2015. The national prevalence rate was estimated to be 0.3% in 2015 with a regional range between 0.2 and over 2% [1]. The epidemic is mostly concentrated in sub-populations who have an elevated risk of HIV transmission. Progress has been made in increasing the number of people tested for HIV and the access to antiretroviral therapy (ART) in recent years. A national comprehensive approach has been recommended to reach further goals [2]. This includes scaling up antiretroviral therapy (ART) regardless of CD_4_ cell count to reduce mortality and improve quality of life [3].

Counselling is an important part of HIV care. Counselling is offered around testing and diagnosing individuals and may be offered at various stages of the disease [4, 5]. Specifically, counselling on medication use addressing adherence may be initiated before and after starting ART. This may include how to access ART, an issue particularly important for resource restricted settings. Counselling may be delivered by health providers or peers. Such counselling has been found to successfully enhance adherence by helping PLHIV to cope with the complexity of ART and stigma associated with taking ART [6–8].

Most research has been done on counselling around the time of diagnosis [4]. Although, as has been highlighted, counselling has been found to be beneficial at later stages of the disease [6–8], less is known about factors preventing PLHIV from attending counselling. Therefore, this study investigated facilitators and barriers to regular counselling attendance of PLHIV patients treated at the Prof. Sulianto Saroso Infectious Disease Hospital (RSPI-SS), a national HIV/AIDs referral hospital in North Jakarta, Indonesia.

## Methods

### Study design, setting and data collection

This was a cross-sectional study using a paper-based questionnaire in outpatients of the RSPI-SS hospital. This study was conducted between July and October 2013. In this hospital, attending counselling was mandatory for PLHIV after collecting ART. The hospital provided either individual counseling by a psychologist or a certified HIV counsellor or attending support groups at the hospital or at an HIV-AIDS Non-Governmental Organization (NGO). The duration of counselling varied depending on the type of counselling/counsellor and the needs of patients. PLHIV were eligible to participate if they were on ART (regardless of duration of therapy), were 18 years of age or older, and signed informed consent. All PLHIV who received their ART from the study hospital in the study period were approached for participation. Patients were recruited by HIV clinic staff i.e., nurses or doctors of the study hospital. No specific training was provided to the recruiters. They informed potential participants when they collected ART in the hospital. Those who were interested were informed about the aim of the study and assured that all information would be kept confidential. Permission was also sought from the participants to access their history of counselling attendance. Once they provided written consent, they were included in the study.

The recruiters gave the questionnaire to the participants. The questionnaire consisted of questions about: age, gender, marital status, travel distance from hospital, education, employment status, income, religion, mode of HIV transmission, co-infection with hepatitis B/C, history of injection drug use and whether any of their family members were HIV positive. It took on average 30 min to complete the questionnaire. Data on the counselling attendance of the last three months were collected from the attendance lists provided by the counsellors. We chose a study period of 3 months, to gain insight into attendance rates of the recent past. Data of the questionnaires and data on counselling attendance were matched and then anonymized.

### Data analysis

The outcome was whether the patient attended any of the three types of HIV counselling (individual counselling, support group sessions at RSPI-SS or NGO support groups) regularly. According to the national guidelines [9], PLHIV have to attend counselling each time they collect ART which is usually supplied once a month. Therefore, we defined regular counselling attendance as attending 3 times counselling in the 3 months prior to recruitment, based on the lists of the counsellors. The outcome was coded as binary (regular counselling attendance = 1, and not regular counselling attendance = 0). We included the following variables in the analysis: gender, age (≤30 years old, 31–40 years old, ≥41 years old), marital status (not married, married, divorced/separated), education (primary or less, secondary, tertiary), employment (full-time, part-time, umemployed), income (< 2.5 mil, 2.5–5 mil, 5–10 mil, > 10 mil, no income), religion (Islamic, Christian, Other), hepatitis B/C co-infection (yes, no), reported history of injection drug user (IDU) (yes, no), reported household member with HIV (yes, no), transmission source (male-to-female, male-to-male, IDU, other), distance from hospital (< 10 km, 10–20 km, > 20 km), years since HIV diagnosis (< 2 years, 2–5 years, > 5 years).

### Statistical analysis

The sociodemographic data were analyzed descriptively. To explore the association between HIV counselling attendance and demographic, medical and behavioural variables we performed a univariate logistic regression analysis to produce unadjusted odds ratios (OR) and corresponding 95% confidence intervals (CI). Any variable with a *p*-value < 0.2 was then tested for collinearity and then subsequently included in a multiple logistic regression model. A backward stepwise selection procedure was used to obtain the final model. Variables with a *p*-value of 0.1 were removed in the process. Adjusted OR and 95% CI were presented for the final model. The Homer-Lemeshow goodness of fit test was used to assess model validity. Data was analyzed using Stata/IC Version 13 (Stata Cop., College Station TX, USA).

## Results

Overall, 1450 PLHIV collected ART in hospital during the study period. Of those, 880 PLHIV agreed to participate (60.7%) and these were included in further analysis. The study participants were predominantly male (71.1%) and worked full-time (54.9%). The majority of participants (81.7%) had completed at least secondary education, and reported to be Muslims (60.9%). Over half (53.2%) self-identified transmission as male-to-female, 30.9% as male-to-male, and the remaining were injection drug users or other/missing. Overall, 78.4% had regularly attended counselling during the study period. Table 1 provides the additional socio-demographic information about the participants.

**Table 1:** Factors associated with regular counselling attendance at Prof. Sulianti Saroso Infectious Disease Hospital, Jakarta, *N* = 880

Characteristic	Sample N (%)	Not regular counselling attendance	Regular counselling attendance	Univariate OR (95% CI)	Multivariate OR (95% CI)
Gender					
Male	626 (71.1%)	148	478	ref	
Female	235 (26.7%)	39	196	1.56 (1.05–2.30)	
Missing	19 (2.2%)				
Age					
≤ 30 years old	221 (25.1%)	37	184	ref	
31–40 years old	487(55.3%)	103	384	0.75 (0.5–1.13)	0.55 (0.32–0.93)
≥ 41 years old	168 (19.1%)	50	118	0.48 (0.29–0.77)	0.30 (0.16–0.55)
Missing	4 (0.5%)				
Marital status					
Not married	311 (35.3%)	74	237	ref	
Married	429 (48.8%)	83	346	1.30 (0.91–1.85)	
Divorced/separated	132 (15.0%)	32	100	0.98 (0.61–1.57)	
Missing	8 (0.9%)				
Education					
Primary or less	32 (3.6%)	6	26	ref	
Secondary	719 (81.7%)	158	561	0.82 (0.33–2.03)	
Tertiary	117 (13.3%)	25	92	0.85 (0.30–2.29)	
Missing	12 (1.4%)				
Employment					
Full-time	483 (54.9%)	148	335	ref	
Part-time	186 (21.3%)	35	168	13.25 (5.74–30.58)	10.71 (4.09–28.02)
Unemployed	203 (23.3%)	6	180	2.12 (1.40–3.20)	1.88 (1.03–3.44)
Missing	8 (0.5%)				
Income					
≤ 2.5 mil	282 (32.0%)	67	215	ref	
2.5–5 mil	276 (31.4%)	43	233	1.69 (1.10–2.58)	
5–10 mil	56 (6.4%)	16	40	0.78 (0.41–1.48)	
> 10 mil	14 (1.6%)	3	11	1.14 (0.31–4.22)	
No income	86 (9.8%)	27	59	0.68 (0.40–1.16)	
Missing	166 (18.8%)				
Religion					
Islamic	536 (60.9%)	131	405	ref	
Christian	272 (30.9%)	41	231	1.82 (1.24–2.68)	1.81 (1.21–2.94)
Other	58 (6.6%)	14	44	1.01 (0.54–1.91)	
Missing	14				
Other factors					
Hepatitis B or C co-infection					
No	782 (88.9%)	155	627	ref	
Yes	89 (10.1%)	33	56	0.42 (0.26–0.67)	0.42 (0.24–0.75)
Missing	9 (1%)				
Reported history of IDU					
No	512 (58.2%)	111	401	ref	
Yes	354 (40.2%)	79	275	0.96 (0.69–1.34)	
Missing	14 (1.6%)				
Reported household member with HIV					
No	756 (85.9%)	178	578	ref	
Yes	124 (14.1%)	12	112	2.87 (1.55–5.36)	3.31 (1.70–6.44)
Missing	0				
Transmission Source					
Male-to-female	468 (53.2%)	92	376	ref	
Male-to-male	39 (4.4%)	18	21	0.29 (0.15–0.56)	0.13 (0.04–0.44)
IDU	354 (40.2%)	77	276	0.88 (0.62–1.23)	
Other mode of transmission	8 (0.9%)	2	6	0.73 (0.15–3.70)	
Missing	12 (1.4%)				
Distance home to hospital					
< 10 km	504 (57.3%)	85	419	ref	
10 km–20 km	211 (24.0%)	60	151	0.51 (0.35–0.75)	
> 20 km	165 (18.7%)	45	120	0.54 (0.36–0.82)	0.56 (0.33–0.93)
Missing	0				
Years since HIV diagnosis					
< 2 years	315 (35.8%)	65	250	ref	
2–5 years	346 (39.3%)	68	278	0.94 (0.64–1.38)	
> 5 years	162 (24.9%)	57	162	0.70 (0.47–1.04)	
Missing	0				

*HIV* human immunodeficiency virus, *IDU* injection drug user, *OR* odds ratio, *CI* confidence interval

PLHIV being above 40 years old (OR = 0.30, 95% CI = 0.16–0.55), and between 31 and 40 years old (OR = 0.55, 95% CI = 0.32–0.93) were more likely to regularly attend counselling compared to being less than 30 years old. Also, being unemployed (OR = 1.88, 95% CI = 1.02–3.44), and having a part-time job (OR = 10.71, 95% CI = 4.09–28.02) were more likely to regularly attend counselling compared to having full-time employment. Having a household member with HIV (OR = 3.31, 95% CI = 1.70–6.44), reporting being Christian (OR = 1.82, 95% CI = 1.12–2.94) were also more likely to regularly attend counselling. Having Hepatitis B or C co-infection, (OR = 0.42, 95% CI = 0.24–0.75), living more than 20 km from hospital (OR = 0.55, 95% CI = 0.33–0.93), and self-identified transmission source male-to-male (OR = 0.13, 95% CI = 0.04–0.44) were less likely to regularly attend counselling (Table 1).

## Discussion

In our cross sectional study, we found that nearly 80% of PLHIV regularly attended counselling. This was less than expected, as every PLHIV who collected ART at the hospital had to attend counselling [9]. Attendance rates in our study are difficult to compare to other studies, since policies and practice of counselling varies across countries considerably [4, 5, 7]. PLHIV who were unemployed or worked part time, have household members with HIV and religion were associated with regular counselling attendance. These results suggest that contrary to other studies there seemed to be no financial constraints among those with part-time or unemployment to attend counselling [6, 10]. But our data also indicate that people working full-time may have time constraints to attend the counselling. Reasons may include that counselling takes place during working hours [11]. Religion was associated with counselling attendance, but we have no explanation for this finding and this has not been investigated previously in Indonesia. Furthermore, older age, having hepatitis B or C, distance to hospital, as well as male-to-male transmission were characteristics of PLHIV not attending counselling regularly. This suggests that older patients, patients with co-morbidites as well as patients living further away from the clinics may need additional support to be able to attend counselling. Possibly, some patients perceived homophobia among health providers which may have kept them from attending counselling [12]. It has been shown previously that attending counselling increases social support and the feeling of being accepted. It also helps PLHIV to cope with fear and the burden of side effects when taking ART [4]. Therefore it is desirable to increase the rate of regular counselling attendance.

Solutions may be to adjust the schedule of counselling to PLHIV needs and increase support to access by referring PLHIV to the nearest community health center which provided ART and counselling. Furthermore, recruiting lay counsellors from the gay community may help this group to attend counselling [13]. Finally, technology may also offer solutions [4, 13]. For example, internet-based counselling for PLHIV may have the advantage of providing more privacy than face-to-face counselling [5]. Pictographs, short message service, and social media, as learning and communication tools may be also used for counselling to explain the concept of ART or respond to questions [12, 14].

### Strength and limitations

With this study we provided useful information on the barriers to attend counselling in an urban area from a large group of PLHIV. Although, we were able to achieve a moderate response rate, PLHIV not regularly attending the clinics and PLHIV refusing to complete the questionnaire may have other barriers to attend counselling. We were able to include a considerable number of factors in our analysis, but there may be other factors associated with counselling attendance which we were not able to capture in a questionnaire [15]. Furthermore, there was some missing data in the questionnaires, but except for income these were between 0 and 2.2% and therefore should not have an impact on our findings. Finally, since we only collected data in an urban context, our results may not be generalizable to rural areas. Further studies should be conducted in other settings and provinces of Indonesia to identify other potential barriers which may affect PLHIV. Since this study is a cross-sectional study, future work should be undertaken to assess counselling attendance and dropout rates over time. Furthermore, the competences of counsellors and stigma have not been measured in this study [16]. Counselling should be done with respect for PLHIV without PLHIV feeling obliged to notify their sexual partners [17]. More work should be done in this area to be able to tailor services better to the needs of PLHIV to improve health outcomes.

## Conclusions

In conclusion, while about 80% of patients regularly attended counselling sessions, services should be reviewed to ensure that they are near home and fit the needs of older patients or patients with co-morbidities and minorities. Tailoring counselling may improve attendance.

## Additional file

Additional file 1: RSPI-SS HIV Counseling Study. (XLSX 281 kb)

## Abbreviations

AIDS: Acquired immune deficiency syndrome
ART: Antiretroviral therapy
OR: Odds ratio
PLHIV: People living with HIV
SMS: Short message service
